# 85. Cryoglobulinaemic vasculitis: a rare manifestation of longstanding rheumatoid arthritis

**DOI:** 10.1093/rap/rky034.048

**Published:** 2018-09-20

**Authors:** Fatemah Baron, Bernadette Lynch

**Affiliations:** Rheumatology Department, Galway University Hospital, Galway, IRELAND

**Introduction:** Prevalence and incidence of rheumatoid vasculitis has declined in recent years due to aggressive rheumatoid arthritis (RA) treatment and the use of biologic agents. It has been reported that approximately 1% of people with longstanding seropositive rheumatoid arthritis develop vasculitis. Chronic RA, male sex, smoking, rheumatoid nodules, high rheumatoid factor (RF) titre, positive anti-CCP and HLA class I and class II genotypes have been associated with an increased risk. Serial studies have disclosed a relationship between clinical evidence of vasculitis and the presence of cryoglobulinemia in RA cohort. Cryoglobulinemic vasculitis is characterised by the depositions of cryoglobulins in tissues, that precipitate at low temperatures and redissolve with rewarming. This condition is associated with a variety of disorders including malignancy, infection and autoimmune diseases. The spectrum of manifestations ranges from mild to severe disease with skin being commonly affected, but, other organs can also be involved. Here, we report a male patient with chronic deforming RA presented with polyarthritis, isolated digital infarcts and sensory neuropathy. The investigation was compatible with a type III mixed cryoglobulinemia.

**Case description:** A 56 year old Caucasian, smoker, male patient with past history of atrial fibrillation and severe seropositive rheumatoid arthritis (RA) presented with a two-week history of lower limb pain, intermittent numbness, and progressive dusky discolouration of toes associated with pain and numbness. On examination, he was found to have chronic RA changes of the hands and bilateral rheumatoid nodules at the elbows, active synovitis of PIPs, MCPs, wrists and ankles and purple discolouration of toes. Peripheral pulses and capillary refill were normal. Rest of the exam was unremarkable. Laboratory workup showed mildly elevated liver enzymes, elevated erythrocyte sedimentation rate and C-reactive protein at 80 and 123 respectively, markedly elevated rheumatoid factor and anticyclic citrullinaed peptide (RF 1900 IU/ml, ACCP 116 U/ml) and normal C3 but low C4. Patient was admitted by the vascular team for blue toes syndrome and he was commenced on iloprost infusion from day one. The differential diagnosis included thromboembolic disease, vasculitis, peripheral vascular disease, Burger’s disease and infective endocarditis. Echo was performed and was unremarkable for any valve vegetation, intra-cardiac thrombus or mass. Furthermore, ankle brachial pressure was within normal range. X-ray hands and feet showed no erosive arthropathy but periarticular osteopenia and ulnar subluxation. CT thorax-abdomen-pelvis showed no sinister abnormalities. CT peripheral angiogram showed three-vessel runoff to the ankles bilaterally. No significant stenosis and no dissection. Transthoracic echocardiogram (ECHO) was reported normal. He was found to have raised IgA, IgG, IgM and type III cyroglobulinaemia detected on two occasions. Hepatitis workup was negative. This clinical picture of chronic seropositive RA with rheumatoid nodules and polyclonal cryoglobulinemia in a smoker led to the diagnosis of cryoglobulinemic vasculitis. Patient was commenced on 1g IV methylprednisolone for three days followed by 60mg prednisolone daily with taper instructions and methotrexate 10mg weekly with folic acid 5mg weekly. He was also prescribed fosavance 70mg weekly, omeprazole 20mg daily, and prophylaxis septrin 960mg three times a week. He then received IV rituximab 1000mg, total of two doses two weeks apart. His initial symptoms greatly improved. Eight weeks later, the patient was still smoking heavily and has stopped taking his medications. He denies any joint pain or swelling. However, he developed worsening purple discolouration of his hands and feet with a necrotic ulcerative lesion on his left first toe. Repeated inflammatory markers showed C reactive protein of 15.3 mg/l ,sedimentation rate of 8 mm/hour, normal complements, rheumatoid factor 467 IU/ml, AntiCCP 32 and no detected cryoglobulins. He was admitted with the working diagnosis of infective ulcer and possible rheumatoid vasculitis based on worsening digital ischemia. Wound swab grew pseudomonas with no evidence of osteomyelitis on imaging. He was prescribed appropriate IV antibiotic course and started again on high dose steroid. The patient showed improvement of his symptoms including ulcerative lesion. On discharge, he was strongly advised to stop smoking and all medications were resumed with tapering instruction for steroid. On a follow-up visit two months later, patient noted improvement of his skin lesions and denied any joint pain or swelling. On examination dry gangrene of digits and healed ulcer. We continued to taper prednisolone gradually. Patient remained on low dose methotrexate due to impaired liver function (X3 upper normal) on higher doses. At his six month follow-up, he denied any symptoms and is compliant to his medications. Three weeks prior to his rheumatology outpatient appointment he was evaluated in emergency department for cough and shortness of breath at rest. Chest x-ray showed hyperexpanded lungs and well circumscribed density in the right upper zone. He was treated with a course of augmentin and clarithromycin for a possible respiratory tract infection. Outpatient CT thorax showed a background emphysema with ill defined opacities in the right upper and lower lobes , and peribronchial wall thickening with multiple nodules in the right lower lobe. All in keeping with inflammatory process suggestive of acute bronchiolitis. He was then evaluated by respiratory team in an outpatient setting. Pulmonary function test was consistent with obstructive pattern suggestive of chronic COPD. Bronchoscopy and washout was done. Fungal, bacterial and tuberculosis workup were negative. Cytology revealed mixed benign respiratory elements and no malignant cells. It was felt that his respiratory symptoms were most consistent with inflammatory process secondary to RA. Patient received his second rituximab cycle as scheduled. Nevertheless, incidental finding of T8 vertebral fracture was noted on CT images. We stopped fosavance and switched him to denosumab 60mg subcutaneous injection every six-months. DEXA scan was requested and steroid was further tapered. Patient had been closely followed for the past eight months. He achieved remarkable laboratory and clinical improvement after two doses of rituximab with no evidence of active joint disease or new extra-articular manifestation of rheumatoid arthritis. He has stopped smoking. His respiratory symptoms have improved remarkably with no evidence of systemic or localised vasculitis affecting any other sites. Digital ulcers have healed and on clinical examination there was a well-demarcated dry gangrene of left big toe that is awaiting auto-amputation. He currently remains on methotrexate 10mg weekly, folic acid 5mg weekly, tapering low dose prednisolone, septrin three times a week, losec 20mg twice daily and 60 mg denosumab injections every six months.

**Discussion:** A number of studies suggest that circulation immune complexes, cryoglobulins (GC), are present in patients with vasculitis and that the underlying vascular injury is mediated by these complexes. Brouet classification is commonly used to classify CG based on rheumatoid factor (RF) binding activity. Type I is usually referred to the presence of isolated monoclonal Ig and it is usually associated with hematologic diagnoses. Type II (mixed cryoglobulinemia) is characterized by a mixture of polyclonal Ig in association with a monoclonal Ig and it is often due to chronic viral infections. Type III is defined as a mixed CGs consisting of polyclonal Ig and it is usually associated with autoimmune disorders. Cryoglubulins are found in a spectrum of disorders and the term is often used to refer to a systemic inflammatory syndrome that affects small-to-medium vessel in which cryoprecipitation manifests as ischemic or occlusive vasculopathy. It is presentation varies from mild to more severe organ involvement. Asymptomatic cryoglobulinemia does not require treatment. Symptomatic secondary cryoglobulinemia is best managed with treatment of the underlying disease, thus therapy varies according to clinical severity and prior treatments. Initial management of cryoglobulinemic vasculitis is with glucocorticoids and immunosuppressive therapy to control symptoms. Rituximab, which is a B-cell depleting therapy, was reported to be an effective therapy in patients with mixed cryoglobulinemia syndrome not associated with chronic HCV infection. Rituximab, as an induction agent, appears to improve articular symptoms in RA with good efficacy and safety to treat patients with systemic vasculitis. It has been reported that 71% of patients would achieve complete remission at six months together with relevant lowering of dose of corticosteroid. However, maintenance therapy is necessary. To the best of our knowledge, there are no randomised trials directly comparing rituximab with cyclophosphamide in these patients. Thus, cyclophosphamide should still be considered a therapeutic option in patients with mixed cryoglobulinemia, especially in life-threatening situations.

**Key Learning Points:** Cryoglobulinemic vasculitis is a rare disorder associated with seropositive rheumatoid arthritis. It is an important differential in patient who presents with digital ischemia. Management should be initiated as soon diagnosis is confirmed with high dose steroid and immunosuppressant drug. Patients should be monitored closely for disease activity as well as medications side effects.

**Disclosure: F. Baron:** None. **B. Lynch:** None.

